# Mitochondrial reactive oxygen species and cancer

**DOI:** 10.1186/2049-3002-2-17

**Published:** 2014-11-28

**Authors:** Lucas B Sullivan, Navdeep S Chandel

**Affiliations:** The Koch Institute for Integrative Cancer Research at Massachusetts Institute of Technology, Cambridge, MA 02139 USA; Division of Pulmonary and Critical Care Medicine, Department of Medicine, The Feinberg School of Medicine, Northwestern University, Chicago, IL 60611 USA

**Keywords:** Mitochondria reactive oxygen species, ROS, Cancer, Metabolism, Antioxidants, Oxidative stress

## Abstract

Mitochondria produce reactive oxygen species (mROS) as a natural by-product of electron transport chain activity. While initial studies focused on the damaging effects of reactive oxygen species, a recent paradigm shift has shown that mROS can act as signaling molecules to activate pro-growth responses. Cancer cells have long been observed to have increased production of ROS relative to normal cells, although the implications of this increase were not always clear. This is especially interesting considering cancer cells often also induce expression of antioxidant proteins. Here, we discuss how cancer-associated mutations and microenvironments can increase production of mROS, which can lead to activation of tumorigenic signaling and metabolic reprogramming. This tumorigenic signaling also increases expression of antioxidant proteins to balance the high production of ROS to maintain redox homeostasis. We also discuss how cancer-specific modifications to ROS and antioxidants may be targeted for therapy.

## Review

### Introduction

Mitochondrial-derived reactive oxygen species (mROS) have increasingly been appreciated to function as signaling molecules that modify cellular physiology. Increased production of ROS has long been observed to be a hallmark of many tumors and cancer cell lines [1]. Early investigations showed that ROS are capable of damaging proteins, lipids, and DNA, and thus it was believed that ROS can be tumorigenic by promoting genomic instability [2]. While high levels of ROS can promote DNA mutations and genetic instability, over the last 20 years a more nuanced view of the role of ROS in cancer has come to light. Specifically, cancer cells generate increased ROS; however, these ROS levels are still below that which cause overt damage. This range of ROS is capable of increasing tumorigenesis by activating signaling pathways that regulate cellular proliferation, metabolic alterations, and angiogenesis. Here, we will focus on the mechanisms of how mROS impact cellular physiology in cancer and the pathways by which cancer cells increase mROS.

#### Reactive oxygen species

**Figure 1:**
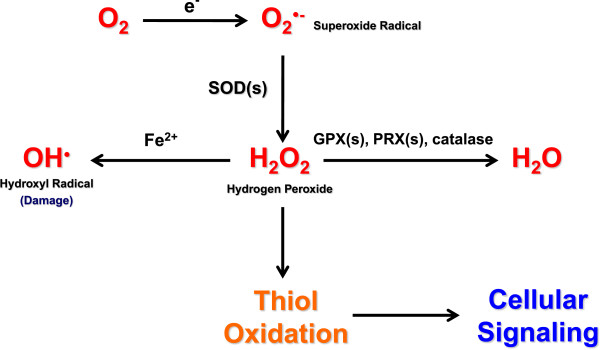
**Production and interconversion of reactive oxygen species.** O_2_^**·−**^ is formed from molecular O_2_ by gaining a single electron from a NADPH oxidase (NOX) enzyme or from electron leak in the electron transport chain of the mitochondria. Superoxide dismutase (SOD) enzymes convert two superoxide molecules into a H_2_O_2_ and a water (H_2_O) molecule. Hydrogen peroxide can undergo Fenton chemistry with Fe^2+^ to form HO^**·**^, which is extremely reactive and can cause cellular damage. Hydrogen peroxide can also modify redox-sensitive cysteine residues to change cellular signaling. Alternatively, hydrogen peroxide can be reduced to water by glutathione peroxidases (GPXs), peroxiredoxins (PRXs), or catalase.

The term reactive oxygen species covers several molecules derived from oxygen that have accepted extra electrons and can oxidize other molecules [3]. Most intracellular ROS are derived from the single electron reduction of oxygen (O_2_) to form the radical superoxide (O_2_^·−^). Two superoxide molecules can then be converted to one molecule of the non-radical ROS molecule hydrogen peroxide (H_2_O_2_) and one water molecule by superoxide dismutases. Hydrogen peroxide can also accept another electron from free Fe^2+^ by the Fenton reaction to become a hydroxyl radical (HO^·^). These three primary forms of ROS have different reactivities that can lead to differential effects on cellular physiology (Figure 1).

Seminal studies in the 1990s demonstrated that the primary signaling ROS molecule is hydrogen peroxide, which can act by inactivating phosphatases to allow for growth factor-dependent signaling [4, 5]. Hydrogen peroxide has the capacity to cross membranes and is significantly more stable than the radical ROS molecules. These attributes allow hydrogen peroxide to encounter susceptible residues on target molecules and display selectivity. One understood mechanism of hydrogen peroxide signaling is through the oxidation of cysteine residues on proteins. Cysteine residues exist in equilibrium between the protonated thiol (Cys-SH) and thiolate anion (Cys-S^−^) forms. Thiolate forms of cysteine are more susceptible to oxidation by hydrogen peroxide to form a sulfenic acid (Cys-SOH) residue [6]. In regulatory cysteine residues this can cause allosteric changes within the protein to modify activity or binding partners. Alternatively, oxidation of active site cysteines can inhibit activity and thus change signaling cascades. The likelihood of cysteine oxidation of a given protein is a combination of solvent accessibility, local hydrogen peroxide concentration, and cysteine pKa [7]. While hydrogen peroxide is the best described signaling ROS molecule, roles for superoxide as an independent signaling molecule have also been described [8]. In addition, other reactive oxidants such as peroxynitrite (ONOO^−^) can form from a reaction between superoxide and nitric oxide (^·^NO). These reactive nitrogen species likely have both overlapping and distinct mechanisms of mediating signaling changes with ROS since they are capable of both oxidizing and nitrating intracellular amino acids. Hydroxyl radicals likely do not play a signaling role since they are generally too reactive to display selectivity in reaction targets.

##### Sources of reactive oxygen species

One major source of intracellular ROS is the NADPH oxidases. NADPH oxidases catalyze the production of superoxide from O_2_ and NADPH. These enzymes were originally described in phagocytes, where they were shown to kill engulfed pathogens by creating locally high levels of oxidative stress [9]. Since this discovery, it has been observed that NADPH oxidase family members are present in many tissues in the body where they are important for non-immune functions as well [10, 11]. The presence of enzymes that specifically produce ROS validates the model that ROS serve a controlled function in the cell, rather than simply acting as toxic by-products. In addition, oncogenes can stimulate NADPH oxidase-dependent ROS production, which has been shown to be necessary for cell proliferation [12]. NADPH oxidases have been detected to be intracellularly localized to many organelles including the plasma membrane, nucleus, mitochondria, and endoplasmic reticulum. Interestingly, the endoplasmic reticulum has recently also been shown to also have NADPH oxidase-independent production of ROS as well [13]. While NADPH oxidases are well-described sources of intracellular ROS, when possible, this review will focus on the mechanisms and consequences of mitochondrial-derived ROS.

The largest contributor to cellular ROS is the mitochondria. It has been estimated that as much as 1% of the total mitochondrial O_2_ consumption is used to produce superoxide [14, 15]. The mitochondria have eight known sites that are capable of producing superoxide [16, 17]. The relative contribution of each of these sites to the total cellular ROS is unclear, however, ROS from complex I, II, and III have all been shown to have effects on cellular signaling [16]. Interestingly, while complexes I and II release ROS into the mitochondrial matrix, complex III has the ability to release ROS to both sides of the mitochondrial inner membrane [18]. Theoretically, releasing ROS to the inner membrane space would allow easier access to cytosolic targets. Consistent with this hypothesis, complex III-derived ROS have specifically been shown to be required for many biological processes including oxygen sensing, cell differentiation, and adaptive immunity [19]. Whether the other sources of mROS have individual or simply contributory roles to the total mROS signaling is unknown.

##### Antioxidant pathways balance ROS levels

Considering that mROS can modify proteins, regulation of the concentration of mROS is crucial for its ability to act as a signaling molecule. Levels of mROS are controlled both at the level of production (discussed below) and by degradation. The SOD proteins (SOD1-3) first convert two superoxide molecules into hydrogen peroxide and water, removing one reactive oxygen species per cycle. Hydrogen peroxide is then further reduced to water by a host of antioxidant enzymes including six PRXs, eight GPXs, and catalase in mammalian cells. PRXs are among the most abundant proteins in cells and have been calculated to degrade most of the intracellular hydrogen peroxide [20, 21]. GPXs also are highly active, although less abundant, and may be an important antioxidant mechanism at higher concentrations of hydrogen peroxide [22]. In the context of ROS signaling, there is accumulating evidence that antioxidant enzymes may be modified in complex ways to facilitate specific ROS signaling events. For example, in response to growth factor signaling membrane-bound PRX1 can be phosphorylated to inhibit degradation of hydrogen peroxide. This results in localized accumulation of hydrogen peroxide and increased growth factor signaling [23]. Similarly, GPX1 activity can be increased by phosphorylation by c-Abl and Arg to protect against high levels of oxidative stress [24]. These examples, as well as the high number of PRXs and GPXs, suggest that the regulation of ROS by antioxidant enzymes may be much more intricate than simply constitutive degradation activity.

**Figure 2:**
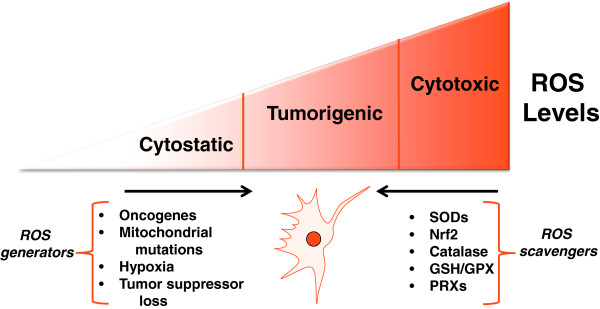
**Balancing ROS generation and ROS scavenging allows cancer cells to remain in the tumorigenic range of ROS levels.** Activation of mitochondrial ROS generation by oncogenes, mitochondrial mutations, hypoxia, or tumor suppressor loss increases ROS signaling to increase tumorigenicity. Tumor cells also express enhanced levels of antioxidant proteins that prevent increased ROS from reaching cytotoxic levels incompatible with growth.

The predominant transcriptional response that increases the production of antioxidant proteins in cancer cells is through the activation of nuclear factor (erythroid-derived 2)-like 2 (NRF2) [25]. Stabilization of the labile transcription factor NRF2 by inhibition of its negative regulator Kelch-like ECH-associated protein 1 (KEAP1) allows it to increase expression of antioxidants including GPXs and glutathione synthesis and utilization genes [26, 27]. One mechanism of NRF2 stabilization is by ROS-mediated oxidation of sensitive cysteine residues on KEAP1 [28–30]. While increased ROS is a common feature in cancer cells, NRF2 has also been shown to be essential for tumorigenesis [31, 32]. It is thus likely that the requirement for NRF2 controls ROS levels in cancer cells to maintain homeostasis. Interestingly, while NRF2 loss inhibited tumor formation, mice deficient for the antioxidant PRX1 have increased ROS and display decreased life span due to hemolytic anemia and development of malignant cancers [33]. Thus, small molecule increases in ROS as a result of removing a single component of the antioxidant response may increase tumorigenesis while complete loss of the antioxidant response pathway, such as in NRF2 knockout mice, results in prohibitively high levels of ROS and decreases tumorigenesis. The distinction between small changes in ROS that promote tumorigenic signaling vs. large changes in ROS that cause oxidative stress to induce cell death is an important factor that will dictate the response to ROS stimuli (Figure 2).

#### Mitochondrial reactive oxygen species regulate signaling pathways

##### ROS enhance phosphoinositide 3-kinase signaling

The phosphoinositide 3-kinase (PI3K) pathway is a central growth factor response pathway that is hyper-activated in many cancers. Activation of this pathway has been shown to increase proliferation, promote survival, and increase cellular mobility [34]. Upon growth factor stimulation, growth factor receptors activate the catalytic subunit of PI3K, p110, through Ras activation or recruitment of the regulatory subunit, p85. Once activated, p110 phosphorylates phosphoinositides (PI) to generate PI (3, 4, 5) P3 (PIP3). PIP3 acts as a signaling lipid by binding to the pleckstrin homology (PH) domain of Akt, causing its localization to the plasma membrane. Akt is then activated by phosphorylation from another PH domain-containing kinase, phosphoinositide-dependent kinase-1 (PDK1). Activation of Akt is an important mediator of the PI3K pathway and leads to increased cell proliferation and suppression of apoptosis. The negative regulator of this pathway, phosphatase and tensin homolog deleted on chromosome ten (PTEN), has constitutive phosphatase activity on PIP3 to convert it to the inactive form, PIP2.

**Figure 3:**
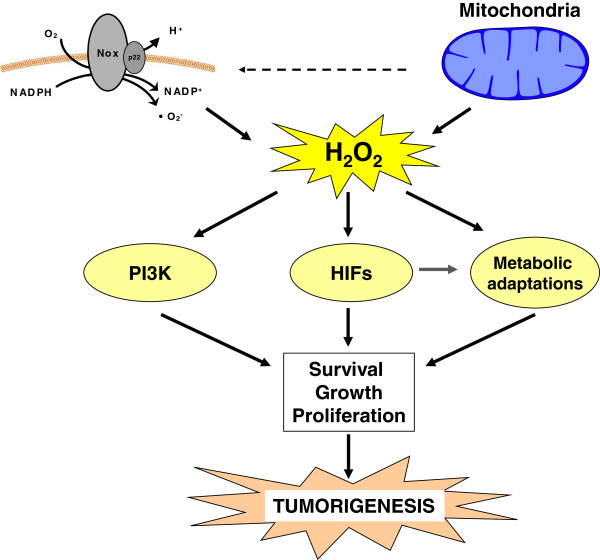
**Reactive oxygen species modify cellular signaling.** Hydrogen peroxide derived from either NOXs or the mitochondria can activate the PI3K pathway, the hypoxia-inducible factor (HIF) pathway, and metabolic adaptations. These modifications are essential to allowing the survival, growth, and proliferation fundamental to tumorigenesis.

The intracellular level of ROS can affect the PI3K pathway. Treatment of cells with exogenous hydrogen peroxide is sufficient to activate Akt [35]. The primary known ROS target in the PI3K pathway is PTEN. ROS have been shown to oxidize the active site cysteine on PTEN (Cys124) resulting in a disulfide formation to another intraprotein cysteine (Cys71). This results in inactivation of PTEN and perpetual activation of the PI3K pathway [36, 37]. In addition to general ROS effects, mROS were specifically shown to inhibit PTEN and activate Akt [38, 39]. Aside from PTEN, ROS have been shown to inhibit other phosphatases, including protein phosphatase 2A (PP2A) and protein tyrosine phosphatase 1B (PTP1B) [40]. PP2A dephosphorylates Akt on threonine 308 and serine 493 resulting in Akt inactivation; however, PP2A dephosphorylation activity is inhibited by hydrogen peroxide [41]. PTP1B also suppresses Akt activity by dephosphorylation but, like PP2A, ROS inhibit PTP1B activity and increase Akt activity resulting in increased anchorage-independent growth [42, 43]. Thus, ROS inhibit phosphatases to dysregulate PI3K signaling resulting in increased Akt signaling and enhanced proliferation and survival (Figure 3).

##### Mitochondrial ROS activate hypoxia-inducible factors

One of the best characterized pathways shown to be responsive to mROS is the hypoxia-response pathway. Hypoxia is a prominent feature of tumor cells *in vivo* due to a mismatch between the high proliferative rate of tumor cells and the ability of the blood supply to provide nutrients including oxygen. Tumor cells activate hypoxia inducible factors (HIFs) to activate a transcriptional network to allow tumor cells to adapt to their diminished oxygen microenvironment. The pathway consists of three hypoxia-sensitive α subunits (HIF1α, HIF2α, and HIF3α) that, upon activation, heterodimerize with the constitutively expressed HIF1β and activate transcription from hypoxia-response elements (HREs) [44]. Under normoxic conditions (21% O_2_), HIFα subunits are rapidly hydroxylated on proline residues by prolyl hydroxylase domain-containing protein 2 (PHD2) which is recognized and targeted for degradation by the von Hippel-Lindau (VHL) E3 ubiquitin ligase pathway [45]. When cells are exposed to hypoxia, PHD2 hydroxylation of HIFα subunits is inhibited leading to HIFα accumulation, heterodimerization, and translocation to the nucleus. The HIF heterodimer interacts with the co-activators p300 and CBP to initiate transcription of hypoxia-response genes from HREs. Appropriately for cells under hypoxia, transcriptional targets of HIFs include genes that promote survival under hypoxia, shift metabolism to increased glycolysis, and activate angiogenesis [46].

Exposure to hypoxia increases mROS to stabilize HIFα subunits. Initial evidence for this mechanism stems from the observation that cells depleted of their mitochondrial DNA (ρ0 cells) are incapable of stabilizing HIFα subunits under hypoxia [47]. ρ0 cells do not exhibit mitochondrial oxygen consumption and do not produce mROS [48]. In addition, treatment of mitochondria-replete cells with the electron transport chain (ETC) inhibitors rotenone, myxothiazol, and stigmatellin can inhibit mROS production and inhibit stabilization of HIFα subunits under hypoxia [49]. In contrast, the ETC inhibitor antimycin A increases mROS and leads to increased HIFα stabilization. Further studies have identified that hypoxia increases the release of superoxide from complex III to the mitochondrial intermembrane space [50]. In complex III, electron transport is mediated by the Rieske-Fe-S protein (RISP), cytochrome b, and cytochrome c1. While the loss of RISP or cytochrome b eliminates mitochondrial oxygen consumption, the loss of RISP eliminates mROS production while the loss of cytochrome b retains mROS production. Importantly, cells depleted of RISP are incapable of stabilizing HIFα under hypoxia, whereas cells depleted of cytochrome b retain their ability to stabilize HIFα [51–54]. This indicates that in hypoxia the increased release of superoxide from complex III is responsible for the inhibition of PHD2 and stabilization of HIFα subunits. How these ROS inhibit PHD2 activity is not fully understood, however, one possibility is that ROS oxidize intracellular Fe^2+^, a cofactor required for PHD2 function [55]. Treatment of cells with mitochondrial-targeted antioxidants blocks the release of mitochondrial ROS and inhibits the stabilization of HIFα subunits under hypoxia [56]. Furthermore, a large chemical screen designed to uncover inhibitors of hypoxic activation of HIFs enriched for mitochondrial inhibitors of complex III [57]. Thus, mROS are both sufficient and required for hypoxic activation of HIFs (Figure 3). Interestingly, suppression of HIF1α by treatment with antioxidants has been shown to inhibit cancer cell proliferation *in vitro* and *in vivo* [58, 59].

##### Mitochondrial ROS modify metabolism

The interplay between ROS levels and cellular metabolism is tightly regulated. Metabolic processes produce ROS, particularly in the mitochondria, thus metabolic fluxes need to be intimately controlled to maintain ROS homeostasis. One important mechanism of metabolic control is through HIF1α. Activation of HIF1α induces expression of glycolysis enzymes and transporters to increase glycolytic flux, as well as increases expression of PDK1 to divert glycolytic carbon away from the mitochondria [60]. In addition, HIF1α induction of NADH dehydrogenase (ubiquinone) 1 alpha subcomplex, 4-like 2 (NDUFA4L2) suppresses complex I activity and mROS [61]. HIF1α has also been shown to induce microRNA-210, which is sufficient to decrease expression of the iron-sulfur cluster assembly proteins ISCU1/2 and decrease mitochondrial oxygen consumption, increase lactate production, and increase ROS [62, 63]. Another method by which ROS can modify metabolism is through activating NRF2. Activation of NRF2 increases synthesis of anabolic enzymes and supports tumor growth by increasing production of NADPH increasing and purine biosynthesis [64]. ROS have also been shown to modify metabolism directly by oxidizing the glycolytic enzyme pyruvate kinase M2 (PKM2). In contrast to its constitutively active splice isoform PKM1, PKM2 is preferentially expressed in cancer cells and is unique due to its ability to be inhibited by a variety of stimuli [65, 66]. Interestingly, ROS have also been shown to inhibit PKM2 activity by directly oxidizing a cysteine residue on PKM2 [67]. Oxidation of this residue was shown to cause increased pentose phosphate pathway flux, increase glutathione levels, and increase proliferation under hypoxia. Importantly, inhibition of pyruvate kinase activity has been associated with increased tumorigenesis *in vi*vo [68].

#### Cancer cells increase mitochondrial reactive oxygen species

##### Tumorigenic mutations increase mROS

**Figure 4:**
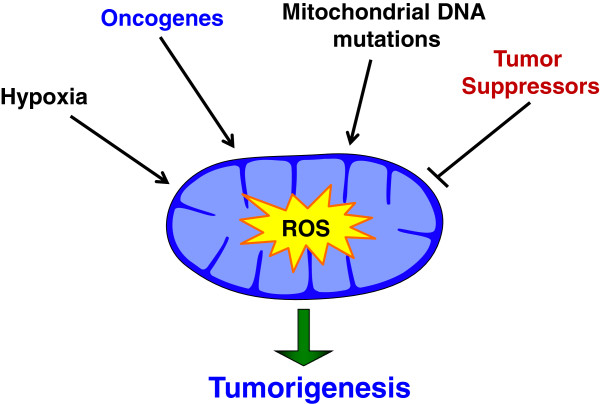
**Pathways that modulate mitochondrial reactive oxygen species.** Hypoxia, activation of oncogenes, mitochondrial DNA mutations, and loss of tumor suppressors have all been shown to lead to a mitochondrial ROS dependent increases in tumorigenesis.

Many cancer cells show increased levels of ROS, and the signaling events and mutations that increase ROS is an area of active research. Several oncogenes have been linked to increased ROS production (Figure 4). Exogenous expression of H-RasG12V has been shown to increase mitogenic activity of 3T3 fibroblasts, and this activity was dependent on increased ROS [12]. In murine embryonic fibroblasts (MEFs) immortalized by a dominant negative p53, expression of Myr-Akt, H-RasG12V, or K-RasG12D conferred increased mROS-dependent soft-agar colony formation [69]. In addition, deregulated expression of Myc has also been shown to modify ROS levels. Exogenous expression of Myc increased ROS production, leading to the transformation in some cells, but ROS induced apoptosis in others [70, 71]. This suggests that the ROS effects may be dependent on cell type, other mutations, and expression level of the oncogene. Interestingly, in mouse models of cancer, activation of physiological expression of K-RasG12D, B-RafV619E, or Myc suppressed steady state levels of ROS [31]. This suppression was shown to be mediated by induction of the NRF2 antioxidant program, and thus it is not clear if oncogenes in this context modify the ROS production or simply decrease steady state ROS by increased expression of antioxidant proteins. Another possibility is that NRF2 expression suppresses the total cell ROS levels, but localized increases in compartmentalized ROS (such as mROS) are maintained to promote tumorigenic signaling.

Several tumor suppressors have been shown to have ROS inhibitory functions. The most common of them, the tumor suppressor p53, known as ‘the guardian of the genome’ is lost or mutated in approximately 50% of cancers [72]. Classically, it has been shown that in response to telomere erosion, oncogene activation, or genotoxic stress that activation of p53 suppresses cancer formation by inducing apoptosis and senescence [73]. However, recent evidence has shown that endogenous expression of p53 with mutations that prevent its ability to cause cell cycle arrest, apoptosis, or senescence still maintains its tumor suppressive function [74]. Interestingly, this mutated p53 retained its ability to control metabolic homeostasis and suppress ROS. In addition, treatment of xenografts with the antioxidant *N*-acetyl cysteine (NAC) suppressed tumor growth in p53 null cancer cells, but not p53 replete cells [75]. These data suggest that p53-mediated tumor suppression may be, in part, due to its ability to suppress ROS (Figure 4).

The sirtuins are a family of NAD^+^-dependent proteins that have been linked to control of metabolic state and cell signaling. Although disputable, several of the sirtuins, including SirT1, SirT2, SirT3, and SirT6, have been implicated to act as tumor suppressors [76]. SirT3, one of the three mitochondrial sirtuins, modulates mitochondrial function by deacetylation of proteins of the electron transport chain, the tricarboxylic acid (TCA) cycle, and antioxidant defense [77]. A survey of human tumors has shown that SirT3 protein expression is significantly decreased in tumors and deletion of at least one copy of SirT3 has been observed in 20%–30% of cancers [78]. Loss of SirT3 expression by genetic knockout or small hairpin RNA (shRNA) increased mROS, while overexpression of SirT3 suppressed mROS [78, 79]. These changes in mROS by SirT3 expression directly correlated with proliferation rate of cancer cells *in vitro* and *in vivo* and could also be modulated with antioxidants.

##### Mitochondrial mutations increase mROS

**Figure 5:**
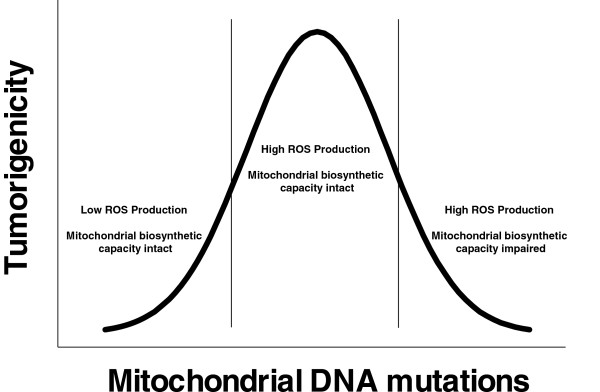
**Heteroplasmic mutations in mitochondrial DNA increase tumorigenesis.** Small amounts of heteroplasmic mutations increase tumorigenicity by increasing mROS levels while maintaining mitochondrial biosynthetic capacity. However, large amounts of mtDNA mutations eventually compromise mitochondrial biosynthetic capacity and will decrease tumorigenicity.

Mutations in mitochondrial DNA (mtDNA)-encoded ETC proteins have been reported in a wide variety of human tumors [80]. Considering cells contain thousands of copies of mtDNA per cell, these mutations typically occur in only a fraction of the total cellular mtDNA, a condition known as heteroplasmy. Heteroplasmic mutations have been observed to be enriched in tumors relative to normal tissue and have been implicated to confer a selective advantage in tumorigenesis [81]. Heteroplasmic mutations in complex I have been shown to increase mROS, increase colony formation in soft agar, and increase tumor formation *in vivo* [82]. Further, reconstitution of complex I activity using the yeast complex I analog NDI1 suppressed mROS, mROS-mediated activation of Akt and HIF1α, and colony formation in soft agar [83]. Perhaps the strongest evidence for the role of heteroplasmic mutations in tumorigenesis comes from a study in which the mtDNA from a poorly metastatic cell line was switched with that of a highly metastatic cell line. Upon acceptance of the new mtDNA, the recipient tumor cells acquired the metastatic characteristics of the opposite cell line [84]. Heteroplasmic mutations in the complex I subunit NADH dehydrogenase subunit 6 (ND6) were shown to increase metastatic potential through increased mROS production and activation of HIF1α. Furthermore, treatment of these cells with the antioxidant NAC inhibited this activity. While relatively low levels of heteroplasmic mutations (10%–60%) increase tumorigenesis, high level heteroplasmy or homoplasmic mutations in mtDNA will likely become detrimental to metabolism and thereby tumorigenicity upon sufficient loss. In support of this model, large levels of heteroplasmy sensitized cells to growth inhibition under low glucose [85]. Cancer cells with mitochondrial mutations resulting in homoplasmic loss of complex I function were unable to form xenografts [86]. In addition, loss of mitochondrial transcription factor A (TFAM), a transcription factor required for mtDNA replication, inhibited tumor formation in an *in vivo* mouse model of K-Ras-driven lung cancer [69]. However, heterozygosity for TFAM caused a mROS dependent increase in intestinal tumorigenesis in an APC^min/+^ mouse model of cancer [87]. Thus, moderate amounts of heteroplasmy may be beneficial for tumorigenesis by increasing mROS while high heteroplasmic mutations or homoplasmic mutations may inhibit tumorigenesis by causing metabolic dysfunction (Figure 5).

Mutations in components of the nuclear-encoded mitochondrial metabolic enzyme succinate dehydrogenase (SDH) have been shown to lead to paraganglioma and pheochromocytoma [88]. The SDH complex is comprised of four subunits (SDHA, SDHB, SDHC, and SDHD) and is the only TCA cycle enzyme that is also a component of the ETC (complex II). Mutations in SDHB, SDHC, and SDHD are commonly associated with cancer formation, whereas mutations in SDHA are rarely associated. Interestingly, given the structure and mechanism of complex II, loss of SDHB, SDHC, and SDHD would allow for acceptance of an electron, but not progression along the ETC, and thus may increase ROS generation. In support of this model, loss of SDHB, but not SDHA increases mROS, HIF1α, and tumorigenicity [89]. In addition, mutations in SDHC are also been associated with increased mROS and tumorigenesis [90]. Thus, loss of components of the SDH complex may, in part, cause tumorigenesis by increasing mROS levels.

In hereditary leiomyomatosis and renal cell cancer (HLRCC), the loss of the TCA cycle enzyme fumarate hydratase (FH) leads to accumulation of the metabolite fumarate and renal cell cancer. FH-deficient cancer cells display pseudo-hypoxia with aberrant activation of HIF1α. Congruent with SDH mutations, this HIF1α activation was also shown to be ROS dependent [91]. However, the mechanism of ROS production is different than SDH mutations. Intracellular thiolate residues on cysteines can undergo a nucleophilic attack on the electrophilic alkene bond of fumarate to produce a ‘succination’ modification [92]. Accumulated fumarate in FH-deficient cells succinates the thiol residue on the intracellular antioxidant molecule glutathione to produce the metabolite succinated glutathione (GSF) [93]. The metabolism of GSF consumes NADPH, the primary reducing equivalent used in ROS detoxification reactions. Thus, GSF reduces overall NADPH antioxidant capacity resulting in increased mROS and HIF1α stabilization. Interestingly, FH-null cancer cells also display hyper-activation of the master antioxidant transcription factor NRF2. While ROS have been shown to stabilize NRF2, FH-deficient cancer cells primarily activate NRF2 by succination and inactivation of KEAP1 [93–95]. Depletion of NRF2 by shRNA in FH-null cells further increased ROS, increased HIF1α stabilization, and decreased proliferation, suggesting that NRF2 suppresses fumarate-mediated ROS to maintain a favorable homeostatic level compatible with proliferation [93].

#### Targeting ROS for therapy

##### Suppressing ROS to inhibit proliferation

ROS contribute to mitogenic signaling, and thus decreasing intracellular ROS levels is an attractive method for inhibiting cancer growth. With this in mind, several large-scale studies have investigated whether supplementation with antioxidant vitamins, including β-carotene and vitamin A or vitamin E can reduce cancer risk in humans. Contrary to the expected result, supplementation increased the risk of cancer in both cases [96, 97]. In agreement with these results, in genetic mouse models of K-Ras- or B-Raf-induced lung cancer, treatment with NAC or vitamin E markedly enhanced tumor growth and accelerated mortality [98]. These results show that the potential use of antioxidants for cancer therapy is complex and needs to be carefully validated before being applied. One possibility for the failure of these antioxidants as cancer treatments is their lack of specificity. Treatment of patients with general antioxidants may modulate many physiological processes that are relevant to cancer growth. For example, the immune system, an important modulator of cancer growth, has been shown to be sensitive to ROS levels [99]. Another possibility is that general antioxidants are differentially effective than targeted antioxidants. Mitochondrial-targeted versions of antioxidants have been shown to be potent inhibitors of cancer cell growth *in vitro* and *in vivo* [69, 100]. Thus, further investigation needs to be considered to determine if targeted antioxidants are a viable method to treat cancer.

Another approach for inhibiting ROS is to decrease production. Decreasing mROS production necessarily involves inhibition of the ETC and thus may not be a practical due to toxicity inherent in inhibiting mitochondrial respiration. However, patients taking the antidiabetic drug metformin have recently been shown to have a reduced risk of cancer incidence and mortality [101]. Metformin has been shown to act as an inhibitor of complex I of the ETC [102, 103]. We recently used a metformin insensitive complex I analog to confirm that the anticancer effect of metformin is primarily mediated by specific inhibition of complex I of cancer cells *in vivo* [104]. Interestingly, we also observed that treatment with metformin suppressed hypoxic activation of HIF1α, indicating that it may also decrease production of mROS under hypoxia. Whether this effect is important for the cancer suppressive effects of metformin requires further investigation. An alternative approach to decrease ROS production is by inhibiting NADPH oxidases. Indeed, loss of NADPH oxidase 4 has been shown to activate apoptosis in pancreatic cancer cells [105]. In addition, inhibitors of NADPH oxidase activity have been shown to have efficacy on mouse models of cancer *in vivo* [106, 107].

##### Increasing ROS to selectively kill cancer cells

**Figure 6:**
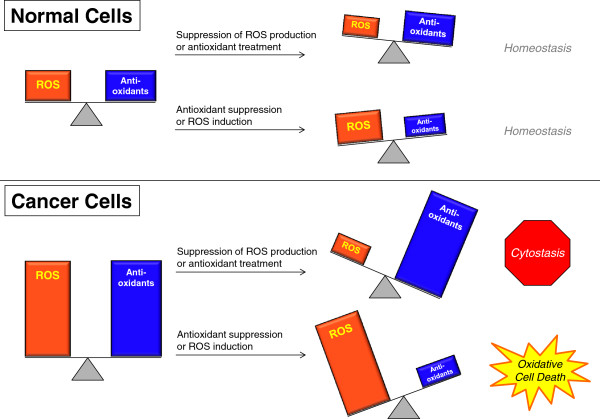
**Targeting cancer cells by modifying ROS levels.** Normal cells have decreased amounts of both ROS and antioxidants relative to cancer cells. Loss of either ROS or antioxidants therefore causes only small changes in ROS homeostasis, leaving cells viable and functional. However, since cancer cells have more ROS and antioxidants, they may be more susceptible to changes in ROS levels. Treatment with antioxidants or prevention of ROS generation will cause cells to lose sufficient ROS signaling to maintain growth. The result is cytostasis and possibly senescence. Alternatively, inhibition of antioxidants or increasing ROS generation will result in excess ROS in cancer cells and cause cancer-specific oxidative cell death.

Considering that cancer cells have increased ROS levels, they may be selectively sensitive to the damaging effects of further increasing ROS. Increasing ROS production specifically in cancer cells is likely difficult to accomplish, although it is one proposed mechanism for how many current chemotherapeutics function [108]. Alternatively, since cancer cells frequently have increased expression of antioxidants to maintain homeostasis, a promising therapeutic approach is to inhibit antioxidants to expose cancer cells to endogenously produced ROS [109]. In support of this model, several small molecule screens identifying compounds that specifically inhibit growth of transformed cells have converged upon glutathione utilization [110–112]. In all cases, treatment with the identified small molecules decreased glutathione levels, increased ROS, and could be rescued by treatment with NAC. In addition, inhibition of antioxidant pathways has also been shown to be effective for inhibiting cancer growth. Genetic knockout of NRF2 inhibited disease progression in mouse models of pancreatic and lung cancer [31, 32]. Inhibition of SOD1 by the small molecule ATN-224 was shown to cause ROS-dependent cancer cell death *in vitro* and decreased tumor burden in advanced K-Ras-driven lung cancers *in vivo* [113]. These recent examples provide further proof of principle that increasing ROS, whether by increasing production or inhibiting antioxidants, is a promising approach for targeting cancer cells (Figure 6). Further research is warranted to determine which components of the antioxidant pathway are selectively essential for tumor growth.

## Conclusions

It is becoming increasingly apparent that ROS play an important role in the biology of tumorigenesis. While several mechanisms have been presented here, the bulk of ROS-mediated signaling targets are largely unknown. However, the frequency of cancer-associated mutations that increase ROS levels suggests that increased production of ROS may be a common output of a large fraction of cancer-associated mutations in oncogenes and tumor suppressors. In addition, the apparent selection for mitochondrial mutations that increase ROS at the detriment of metabolic flexibility suggests that ROS are strongly selected for in these cancer cells. An emerging model is that cancer cells increase the production of ROS to activate localized pro-tumorigenic signaling but balance the increased ROS with elevated antioxidant activity to maintain redox balance. As with all studies in cancer, the final goal will be to design therapeutics that can take advantage of these discoveries. Both the suppression of ROS to prevent activation of pro-tumorigenic signaling pathways and the exacerbation of ROS by disabling antioxidants to induce cell death represent promising approaches in this regard. Future work is needed to better understand ROS-targeted pathways. In addition, future studies need to determine what sources of ROS and what specific antioxidants are required for homeostasis. With this knowledge, we can better understand cancer biology and design novel therapeutics to specifically treat cancer cells.
